# NETWORK ANALYSIS OF OBJECTIVE AND SUBJECTIVE INDICATORS OF SOCIAL ISOLATION AND FUNCTIONAL LIMITATIONS

**DOI:** 10.1093/geroni/igad104.1891

**Published:** 2023-12-21

**Authors:** Chuwen Zhong, Linh Dang, Aparna Ananthasubramaniam, Briana Mezuk

**Affiliations:** School of Public Health, University of Michigan- Ann Arbor, Ann Arbor, Michigan, United States; University of Michigan, Ann Arbor, Michigan, United States; University of Michigan, Ann Arbor, Michigan, United States; University of Michigan, Ann Arbor, Michigan, United States

## Abstract

Functional limitations, including decreased physical performance and increased limitations in daily activities, are common among older adults and associated with both objective and subjective social isolation. However, the dynamic interactions between and within different indicators of functional limitations and social isolation are unclear. This study employed network analysis to graphically depict and explore the relationships between various objective and subjective indicators of functional limitations and social isolation. Data came from the 2014/2016 waves of the Health and Retirement Study (n=9,076, mean age =67.25,52.32% female, 80.17% non-Hispanic White). Functional limitations were measured by objective physical performance (grip strength, tandem balance test) and self-reported limitations in daily activities. Objective social isolation was based on marital status, living arrangement, and network composition (children, other family members, and friends); subjective social isolation was indicated by the UCLA loneliness scale. Separate networks were constructed for men and women. Centrality indices, including strength, betweenness centrality, and eigenvector centrality, were computed to quantify the influence of each item. Subjective functioning and isolation items were strongly correlated but had weaker interconnections with one another. Strength and eigenvector centrality indicated that subjective functioning items had high degrees of influential interactions. Subjective social isolation items had high values of betweenness centrality, suggesting important bridging roles of these constructs. Subjective social isolation items had a more influential role in the network of women than men. Findings suggest opportunities for interdisciplinary interventions to address the multifactorial relationship between functional limitations and social isolation among older adults.

